# Supplementary material for: Incipient speciation, high genetic diversity, and ecological divergence in the alligator bark juniper suggest complex demographic changes during the Pleistocene
Source: PeerJ. 2022 Jul 26;10:e13802. doi: 10.7717/peerj.13802 (PMC9336613; doi:10.7717/peerj.13802)
Supplement: Supplemental Information 1 [file peerj-10-13802-s001.docx]

**Supplementary information**

**Appendix S1. Primers**

**Table S1.1**. Primer sequences used.and references.

| Marker | **Primer** | **Sequence** | **Reference** |
| --- | --- | --- | --- |
| *trnL-trnF* | trnLUAA  trnFGAA | CGAAATCGGTAGACGCTACG  ATTTGAACTGGTGACACGAG | (Taberlet et al., 1991) |
| *Lfy* | Lfy5J F  Lfy4J R | ATGTTCAGCACGTCGCAAAGG  TTGTCGATATGACCTACACCAG | (Willson et al., 2008) |
| *Myb* | Myb F  Myb R | TAACCAGCTTTGCCCTCAG  ATACAATTCGCGGCTACCATA | (Tsumura et al., 1997) |
| *Myb-J* | MybJ F  MybJ R | ACGCTATYAAGAACCACTGGAATTC  GCACTCAATCCATTCATGTAAGAGC | Designed in this study from Myb sequence. |

**Appendix S2. Model construction and evaluation.**

To map the current potential distribution of each of *J. deppeana’*s varieties, as well as the extent of their suitability areas under past climatic conditions, i.e. Last Interglacial (LIG; ~ 130,000 yr ago) and the Last Glacial Maximum (LGM; ~20,000 yr ago), we use species distribution modeling (SDM) using the pseudo-absence algorithm Maxent 3.4.1 (Phillips et al., 2017)⁠ and projected them into the corresponding available climatic layers for each period.

We compiled a dataset of georeferenced records for *J. deppeana* with records from the Global Biodiversity Information Facility (GBIF.org on 23 July 2018. GBIF Occurrence Download https://doi.org/10.15468/dl.ymwero) as well as collection records from the MEXU herbarium (IBUNAM, Mexico City) and grouped them into subsets according to variety distribution. Almost every variety described has its particular distribution range except for var. *patoniana* and var. *sperryi*, which are known from a small number of localities inside the distribution ranges of varieties *deppeana* and *robusta* (Adams, 2014)⁠, therefore were not considered. Moreover, variety *deppeana* has a discontinuous distribution and its range in the USA has been proposed to belong to a different variety (i.e. *Juniperus deppeana* var. *pachyphlaea* (Torr.) Martinez) (Farjon & Filer, 2013)⁠ so it was divided into two subsets (North and South) to further investigate this alternative classification. Groups were then assembled as follows: var. *gamboana* (Chiapas highlands), var. *deppeana* (South) (Central México, at the TMVB), var. *deppeana* (North) (mainly in Arizona, New Mexico, and Texas, with distribution patches in northeast Mexico), var. *robusta* (mainly across the SMOcc mountains) and f. *zacatecensis* (mainly in Zacatecas and East Durango). To avoid spatial autocorrelation we detected and deleted all duplicated records and additionally thinned the database with a filtering distance of 5 km (see raster resolution below). Final records of each group were further divided, using 75% as the training dataset and 25% as the testing dataset for the model construction and evaluation.

We explored climatic variability in the unsorted dataset with the 19 bioclimatic variables available at WorldClim (at a 30arc-sec resolution) (Hijmans et al., 2005). We discarded highly correlated variables (Pearson’s r<0.85 and r>-0.85) as a manner to deal with multicollinearity and finally selected 10 non-correlated variables based on their contribution to total variability. This set of variables was used to build models for each group and the extent area was selected for each group taking into account their dispersal ability. Juniper seeds are prone to be dispersed by birds and little mammals, however, little is known about their true dispersal capacities (Adams, 2014; Farjon & Filer, 2013)⁠, therefore we approximated this area using a bounding box extent of 2 degrees from the training records.

With the R package WALLACE (Kass et al., 2018)⁠ we tested different levels of model complexity to find the best settings to build the final models for each group. For this analysis, we used only training data and tested among the available feature classes (i.e. L, linear; Q, quadratic, H, inge; and P, product) and a range of regularization multipliers from 0.5 to 2.0, with a 0.25 step for a total of 36 evaluated models for each group. We used a 4 block partition for model evaluation and selected the best model based on the corrected Akaike information criterion (AICc) and the AIC weight. Once settings were defined, final models were built using the average model of 20 cross-validation replicates with logistic output in MAXENT. Final models were evaluated using the test dataset to calculate the continuous Boyce Index in the R package ‘ecospat’ (Di Cola et al., 2017)⁠. We used the continuous Boyce Index (Boyce et al., 2002)⁠ because it is an appropriate threshold-independent metric for model evaluation when using presence-only data. It varies from -1 to 1, where models with values above 0 are considered better than random (i.e. are consistent with the distribution data), models with values of 0 are considered no better than random, and values below 0 indicate incorrect prediction models (Hirzel et al., 2006)⁠.

The SDM of each group was projected onto the corresponding bioclimatic layers for each period. We used bioclimatic layers for the LGM for two general circulation models (GCM), the Model for Interdisciplinary Research on Climate (MIROC-ESM) (Watanabe et al., 2011) and the Community Climate System Model (CCSM4) (Gent et al., 2011) retrieved from the *Climatologies at high resolution for the earth’s land surface areas* (CHELSA; https://chelsa-climate.org/last-glacial-maximum-climate) database at a 2.5 min resolution. Bioclimatic layers used for the LIG (Otto-Bliesner et al., 2006)⁠ were retrieved from PaleoClim ([http://www.paleoclim.org](http://www.paleoclim.org/)) (Brown et al. 2018) at a 2.5 min resolution. Final maps were constructed converting the SDM of each period (i.e. current conditions, LGM and LIG) into binary maps using a 10-percentile of the training records threshold, and areas with climatic stability between current conditions and the LGM were defined in terms of each group’s predicted suitability.

**Figure S2.1.** Principal component analysis of the selected climatic variables. The distribution of each record is shown along the first and second principal component, which together account for more than 60% of total variation.


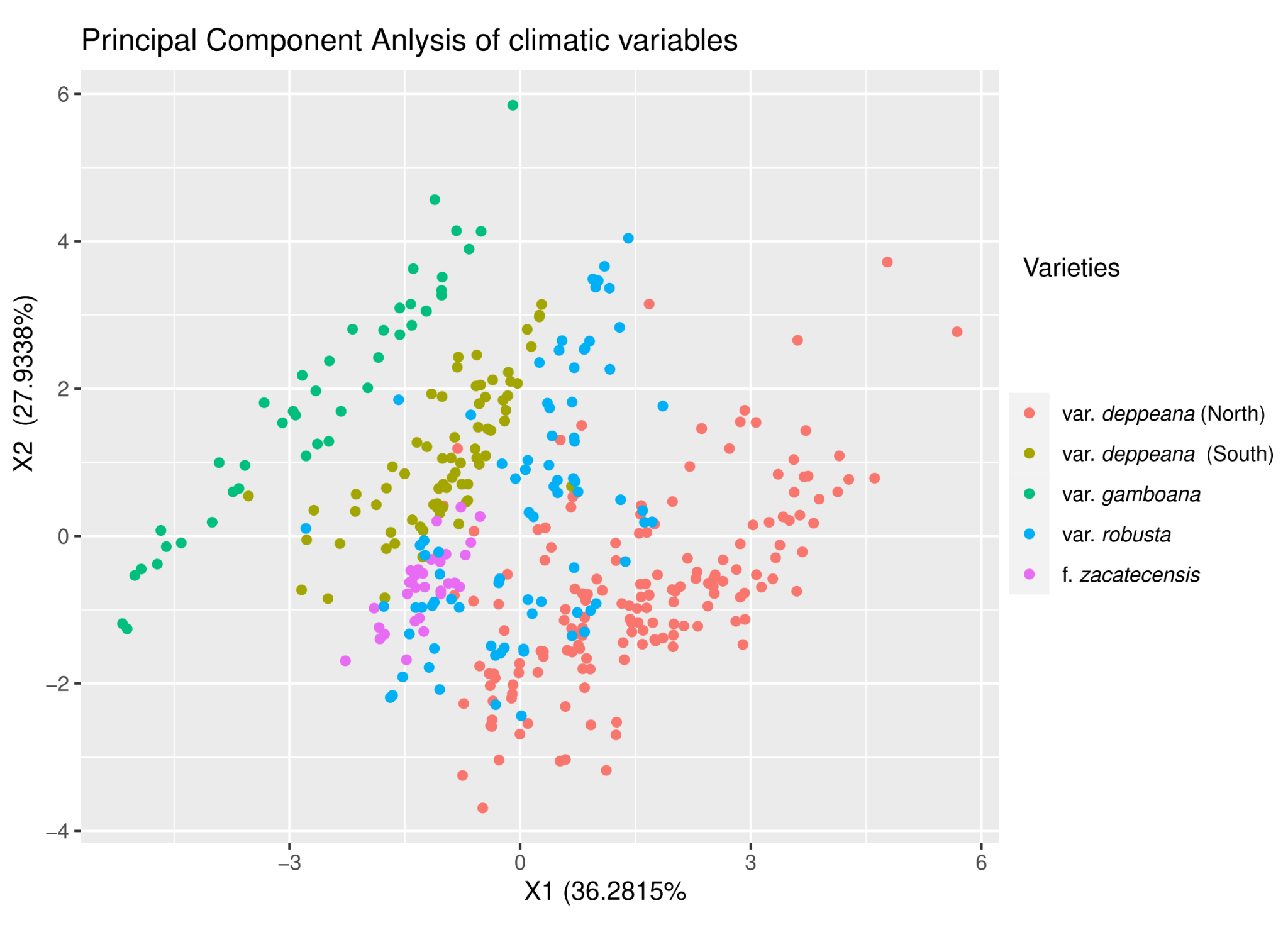


**Figure S2.2.** Heatmap of correlation between climatic variables. The correlation coefficient between each pair of CHLESA climatic variables is shown.


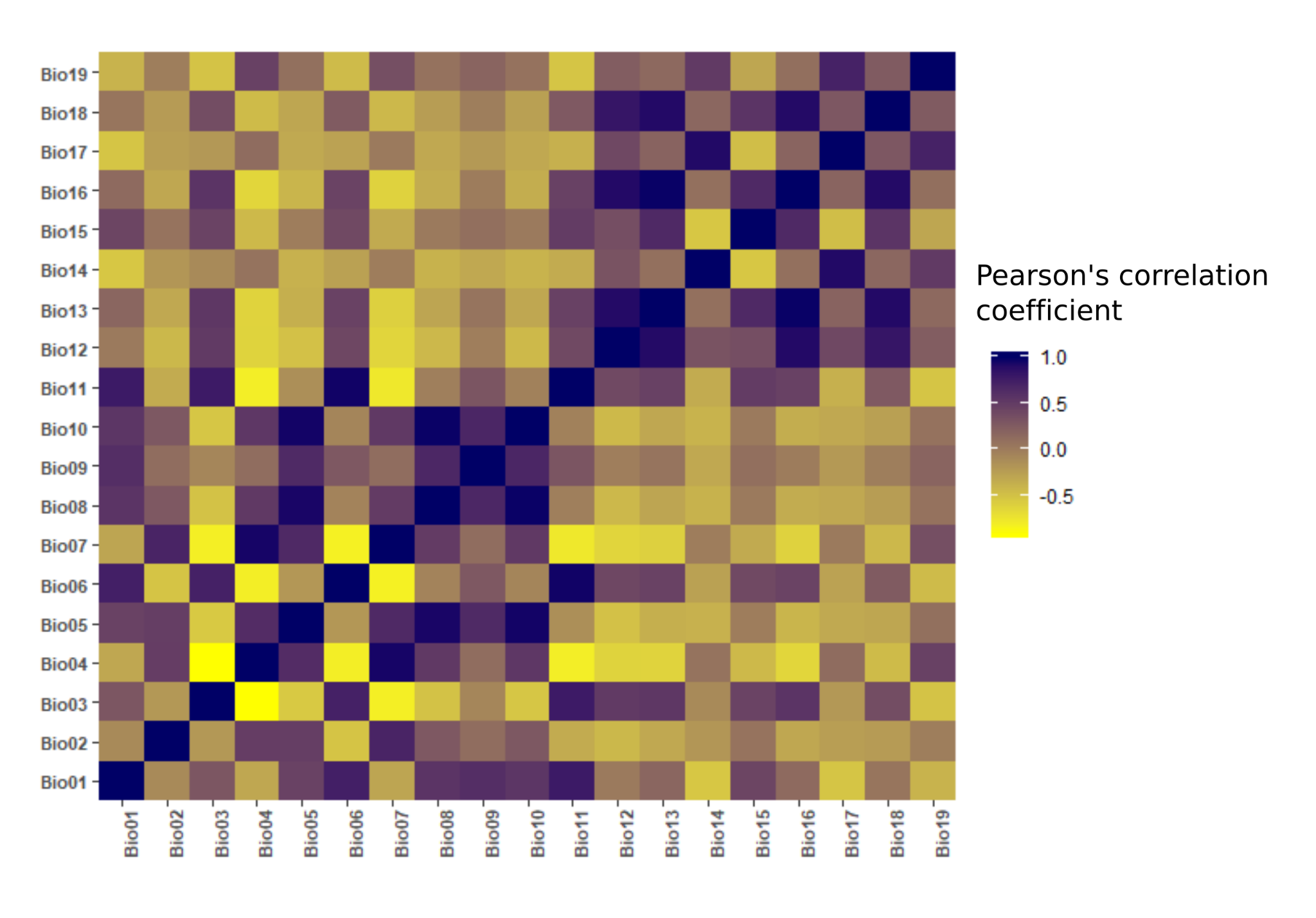


**Appendix S3** Summary information on genetic markers.

**Table S3.1.** Summary information for each marker sequenced. N, number of sequences. PIS, parsimony informative sites. Nuclear loci (i.e. *Lfy* and *Myb*) have 2 sequences per individual.

| Marker | **Longitude (pb)** | **N** | **Invariant sites** | **Polymorphic**  **sites** | **PIS** | **Sites with indels** |
| --- | --- | --- | --- | --- | --- | --- |
| *trnL-trnF* | 302 | 105 | 284 | 4 | 2 | 14 |
| *Lfy* | 775 | 154 | 650 | 59 | 40 | 66 |
| *Myb* | 607 | 184 | 568 | 37 | 21 | 2 |

**Appendix S4.- Genetic structure**


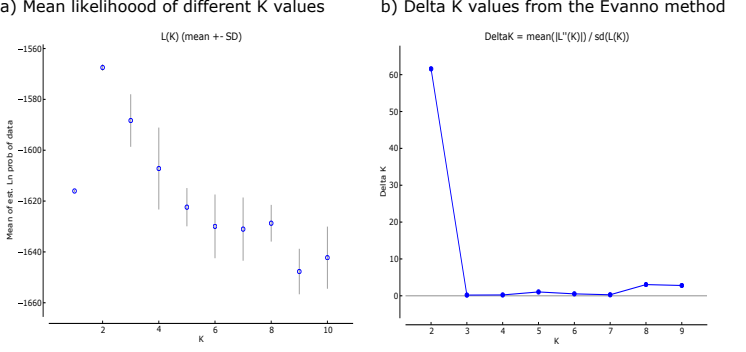
**Figure S4.1.** Structure likelihood values. a) Mean likelihood for different K values; b) Delta K values as estimated with the Evanno method.


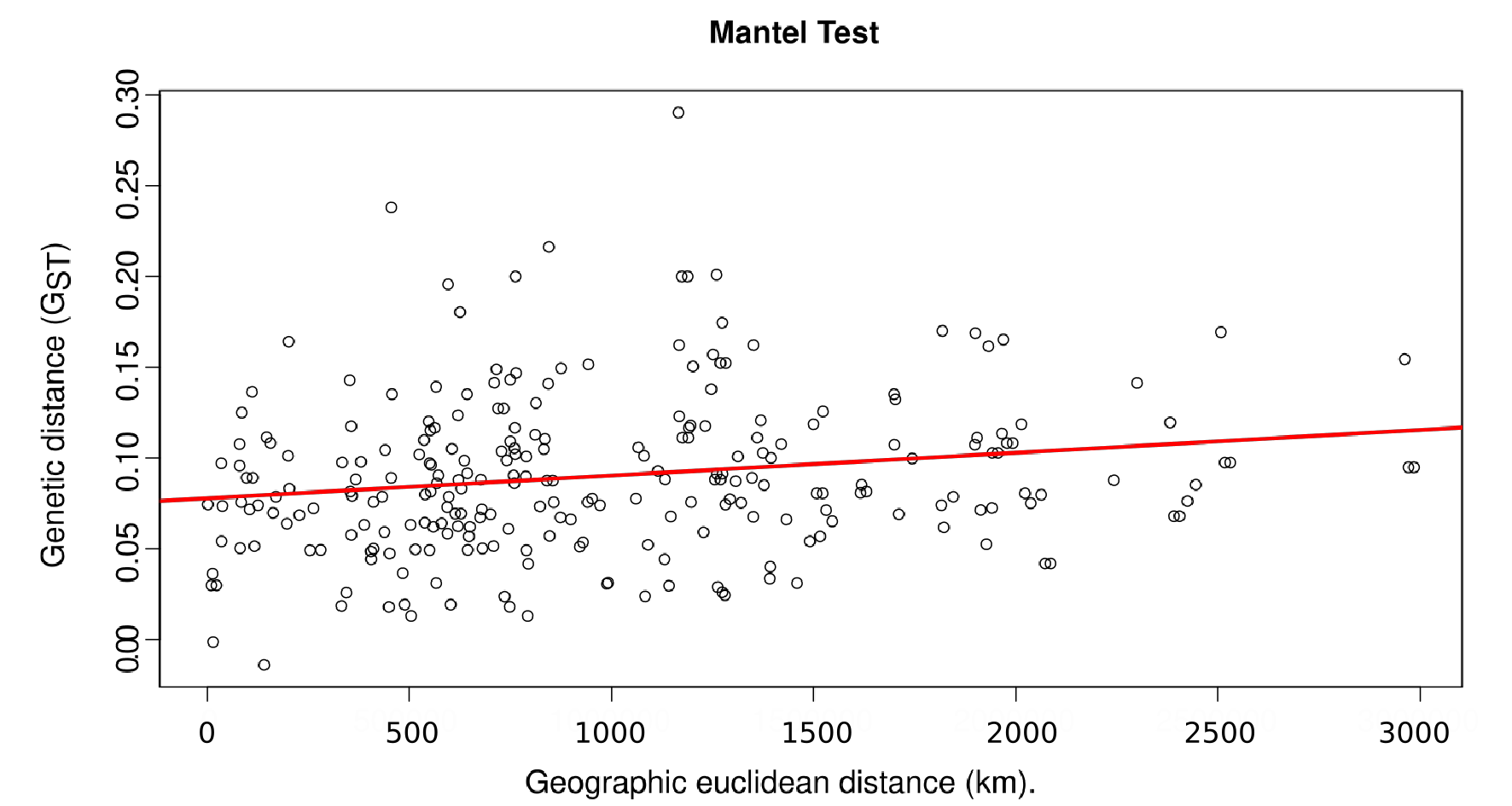


**Figure S4.2.** Mantel test. Correlation between genetic and geographic distances in the sampled populations of *Juniperus deppeana*.

**Appendix S5**


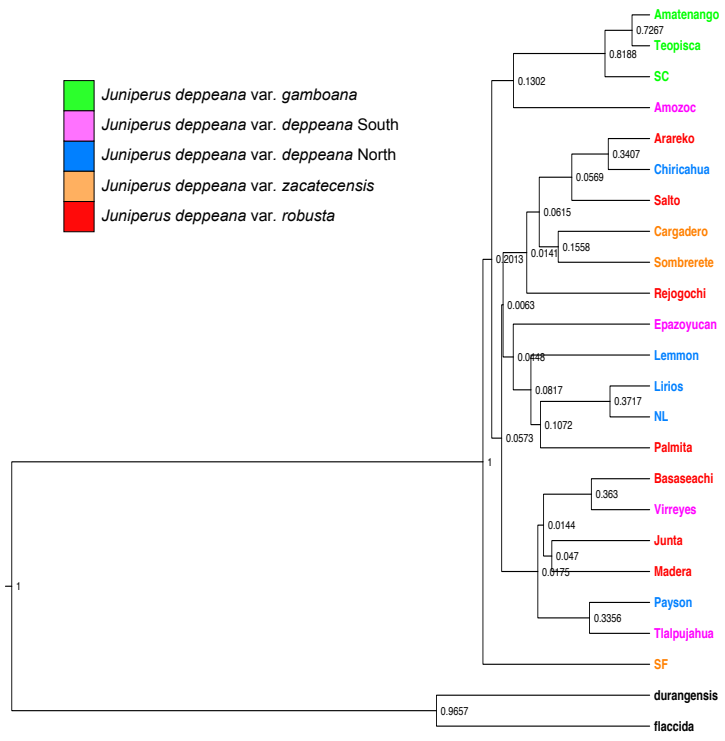


Figure S5.1. Multilocus phylogenetic reconstruction using StarBeast. Terminals are populations.
